# Low BMI increases the risk of metabolic acidosis in children with dehydration

**DOI:** 10.3389/fnut.2026.1737321

**Published:** 2026-05-25

**Authors:** Juraj Stanik, Simona Tarnokova, Lenka Langerova, Tomas Dallos

**Affiliations:** 1 Department of Paediatrics, Medical Faculty of the Comenius University and National Institute for Children’s Diseases, Bratislava, Slovakia; 2 Department of Metabolic Disorders, Institute of Experimental Endocrinology, Biomedical Research Center, Slovak Academy of Sciences, Bratislava, Slovakia; 3 Department of Laboratory Medicine, National Institute for Children’s Diseases, Bratislava, Slovakia

**Keywords:** BMI, children, dehydration, metabolic acidosis, risk factors

## Abstract

**Background:**

Metabolic acidosis (MA) is one of the key factors determining the need for inpatient care in children with dehydration. This study aimed to identify risk factors for the development of MA in otherwise healthy children presenting with dehydration.

**Methods:**

A total of 328 children (50.9% girls, aged 3 months to 15 years) without a history of relevant chronic disease and hospitalized with acute dehydration were analyzed retrospectively. Selected anamnestic, clinical, and anthropometric factors potentially associated with MA were evaluated. MA was defined as a blood pH < 7.36 and a serum bicarbonate (HCO₃^−^) < 22 mmol/L.

**Results:**

Metabolic acidosis occurred in 117 children (35.6%, mean age 4.93 ± 2.14 years). In multivariate logistic regression analysis, MA was independently associated with a higher degree of dehydration (*p* < 0.001), absence of diarrhea (*p* = 0.001), and zero oral intake (*p* = 0.034), and negatively associated with BMI (*p* = 0.024). Neither age nor body weight was significantly associated with MA.

**Conclusion:**

Low BMI and absence of diarrhea were identified as additional risk factors, alongside dehydration severity, for the development of MA in children with acute dehydration.

## 1 Introduction

Dehydration is a common condition among children, even in developed countries, frequently occurring in conjunction with metabolic acidosis (MA) (1). MA is most often associated with acute illnesses, particularly acute gastroenteritis or vomiting (1). Two main pathophysiological mechanisms contribute to the development of MA during acute illness in children, includes accumulation of acids (mainly ketones) and loss of bases (sodium bicarbonate).

In children with prolonged fasting or reduced oral intake, decreased blood glucose (2) and plasma insulin levels, together with elevated counter-regulatory hormones, activate hormone-sensitive lipase and lipolysis (3). This results in increased plasma levels of free fatty acids (FFAs), enhanced *β*-oxidation, and subsequent ketogenesis (4). Ketone bodies, especially β-hydroxybutyrate, cause an increased anion gap and a secondary decrease in bicarbonate. In children with diarrhea, bicarbonate loss through stool may also lead to acidosis, but the anion gap remains normal.

MA often determines the need for intravenous rehydration therapy and hospitalization, and thus affects prognosis and further management (5). Known risk factors for MA include a greater degree of dehydration (5) and a younger age (6). As body composition and energy reserves may influence metabolic responses to fasting and dehydration, this study hypothesized that lower Body Mass Index (BMI) and BMI standard deviation score (BMI-SDS) could be associated with an increased risk of MA. Therefore, this study retrospectively analyzed risk factors for MA in otherwise healthy children and adolescents hospitalized with dehydration due to vomiting or reduced oral intake.

## 2 Methods

### 2.1 Study participants

A total of 328 children (50.9% girls) aged 3 months to 15 years, admitted for acute dehydration to the Department of Paediatrics at the National Institute for Children’s Diseases in Bratislava over a period of 10 years (2014–2023), were included in the study. Children with relevant chronic metabolic, endocrine, gastrointestinal, nephrological, or genetic diseases were also excluded.

### 2.2 Clinical evaluation

Baseline data included age, gender, duration and frequency of vomiting, duration of reduced oral intake (partial or complete), body temperature, and presence of diarrhea. The dehydration severity was assessed by a pediatrician according to clinical signs, including mild (no clinical signs), moderate (interstitial dehydration), and severe (signs of circulatory centralization) (7).

### 2.3 Anthropometry

Anthropometric measurements were obtained at admission by trained nurses following standardized protocols. BMI was calculated as weight (kg) divided by height squared (m^2^). SDS for BMI and height were calculated using local reference data (8).

### 2.4 Biochemical measurements

Blood samples were collected at the time of hospital admission and analyzed as fresh routine samples in the institutional laboratory. Capillary blood was used for assessment of pH, blood gases, and glucose. Serum analyses included urea, creatinine, albumin, uric acid, sodium, potassium, calcium, phosphorus, and chloride levels. Ketone bodies were determined in the first urine sample or as serum *β*-hydroxybutyrate.

Metabolic acidosis was defined as pH < 7.36 and serum bicarbonate (HCO₃^−^) < 22 mmol/L. The HCO₃^−^ threshold of <22 mmol/L is consistent with previously published clinical studies in pediatric populations and reflects commonly used biochemical criteria for metabolic acidosis in acute illness (9). Specifically, several pediatric studies define metabolic acidosis as a pH < 7.35 in combined with a serum HCO₃^−^ < 22 mmol/L in children with dehydration or critical illness (9, 10). The pH cutoff was based on the lower limit of the local laboratory reference range.

The anion gap (AG) was calculated as: AG = sNa – (sCl + sHCO₃^−^). An AG > 16 mmol/L was considered elevated.

### 2.5 Statistical analysis

Metric data were tested for normality using the Shapiro–Wilk test. Normally distributed data are presented as mean ± SD; non-normally distributed data (e.g., BMI and creatinine) are presented as median (interquartile range, IQR 25–75%). Confidence intervals for proportions were calculated using the Wilson/Brown method.

Between-group comparisons were performed using the *t*-test (for normally distributed data), Mann–Whitney *U*-test (for non-normally distributed data), or Fisher’s exact test (for categorical data).

To control for multiple testing in selecting independent variables for multivariate analyses (including sensitivity analyses), the Bonferroni correction was applied. Based on 30 tested associations, the threshold for statistical significance for variable entry into multivariate models was set at *p* < 0.0017 (0.05/30). For all other analyses, a two-sided *p*-value of <0.05 was considered statistically significant.

The multivariate logistic regression models were constructed using forward stepwise selection to identify independent predictors of MA.

To assess the robustness of the association between BMI and MA to the diagnostic definition used, several sensitivity analyses were performed. First, the multivariate logistic regression models were repeated using alternative definitions of MA based on different pH and HCO₃^−^ cutoffs. Second, acid–base parameters (such as pH, serum HCO₃^−^, and anion gap) were analyzed as continuous dependent variables using the multivariate linear regression models constructed using forward stepwise selection. Finally, all analyses were repeated using dehydration-adjusted BMI (AdjBMI).

BMI and AdjBMI were log10-transformed prior to analysis due to non-normal distribution. All multivariate models were adjusted for the degree of dehydration, the presence of diarrhea, and zero oral intake at admission.

Analyses were conducted using SPSS v27 (IBM, NY, United States) and GraphPad Prism 7 (GraphPad, CA, United States).

### 2.6 Ethics

The study was approved by the Ethics Committee of the National Institute for Children’s Health in Bratislava, Slovakia, and conducted in accordance with the Declaration of Helsinki.

## 3 Results

### 3.1 Clinical characteristics

Among 328 otherwise healthy children (mean age 4.32 ± 3.05 years; 50.9% girls) hospitalized for acute dehydration, 117 (35.6%) met the criteria for metabolic acidosis (pH < 7.36, HCO₃^−^ < 22 mmoL/L). Cohort characteristics are summarized in Table 1.

**Table 1:** Basic characterisation of the study group and comparison of groups with and without metabolic acidosis.

Parameter	Together	No metabolic acidosis	Metabolic acidosis	*p*-value
*n*	328	211	117	
Gender (% girls)	50.9	48.8	54.7	0.356
Age (years)	4.32 ± 3.05	3.97 ± 3.41	4.94 ± 2.14	0.002
Diarrhea (% yes)	20.7	31.8	0.9	<0.001
Oral intake (% zero oral intake)	41.1	32.2	56.4	<0.001
Degree of dehydration**	1.5 ± 0.547	1.35 ± 0.53	1.79 ± 0.45	<0.001
Height (cm)	103.16 ± 22.64	99.65 ± 25.19	109.52 ± 15.21	<0.001
Height SDS	−0.15 ± 0.99	−0.13 ± 1.04	−0.18 ± 0.88	0.628
Weight (kg)*	15.45; 11.5–20	13.7; 9.8–20	16,9; 14.5–20	<0.001
Weight SDS*	−0.62; −1.12–0.11	−0.49; −1.1–0.2	−0.76; −1.17 to −0.15	0.056
BMI (kg/m^2^)*	14.9; 13.88–16.39	15.5; 14.23–16.98	14.15; 13.4–15.5	<0.001
BMI SDS*	−0.7; −1.25–0.11	−0.57; −1.13–0.3	−0.96; −1.34 to −0.23	0.004
Capillary pH	7.39 ± 0.08	7.43 ± 0.05	7.3 ± 0.04	<0.001
Capillary pCO2 (kPa)	3.43 ± 0.72	3.62 ± 0.69	3.08 ± 0.64	<0.001
Capillary base excess (mmol/L)	−7.74 ± 5.07	−4.74 ± 3.19	−13.15 ± 2.88	<0.001
Capillary HCO3-(mmol/L)	15.83 ± 4.61	18.24 ± 3.53	11.49 ± 2.77	<0.001
Anion gap (mmol/L)	18.1 ± 5.87	15.67 ± 5.12	22.5 ± 4.4	<0.001
Serum sodium (mmol/L)	137.77 ± 3.61	137.67 ± 3.82	137.95 ± 3.18	0.508
Serum potassium (mmol/L)	4.61 ± 0.54	4.53 ± 0.55	4.75 ± 0.47	<0.001
Serum chloride (mmol/L)	103.84 ± 4.4	103.77 ± 4.62	103.96 ± 4	0.709
Serum calcium (mmol/L)	2.51 ± 0.13	2.49 ± 0.14	2.53 ± 0.12	0.038
Serum phosphorus (mmol/L)	1.67 ± 0.31	1.6 ± 0.33	1.76 ± 0.26	<0.001
Serum magnesium (mmol/L)	0.88 ± 0.1	0.88 ± 0.1	0.87 ± 0.1	0.567
Glycaemia (mmol/L)	4.28 ± 1.4	4.77 ± 1.33	3.39 ± 1.05	<0.001
Serum urea (mmol/L)	5.12 ± 2.07	4.39 ± 2.01	6.38 ± 1.52	<0.001
Serum creatinine (μmol/L)*	35; 28–42	31; 25–38	39; 34.25–43.75	<0.001
Serum uric acid (μmol/L)	433.6 ± 187.31	340.13 ± 134.74	590.76 ± 155.65	<0.001
Serum betahydroxybutyrate (mmol/L)	4.39 ± 1.76	2.95 ± 1.4	5.16 ± 1.44	0.002
Serum T-cholesterol (mmol/L)	3.93 ± 0.93	3.66 ± 0.89	4.33 ± 0.87	0.063
Serum triglycerides (mmol/L)	0.89 ± 0.44	0.95 ± 0.52	0.81 ± 0.33	0.426
Serum albumin (mmol/L)	45.49 ± 4.97	43.75 ± 4.75	48.19 ± 4.04	<0.001

Normally distributed data are expressed as the mean ± standard deviation. Non-normally distributed data* are presented as the median and interquartile range. Abbreviations: BMI – body mass index, SDS – standard deviation score, **mild = 1, moderate = 2, and severe = 3. *p*-value for the difference between groups of children with and without metabolic acidosis.

### 3.2 Characteristics of metabolic acidosis

Of the 117 children with MA, 105 (89.7%) had an elevated anion gap, and all with available data (*n* = 92) showed elevated ketone levels in blood or urine (Table 1).

### 3.3 Anamnestic factors

**Figure 1:**
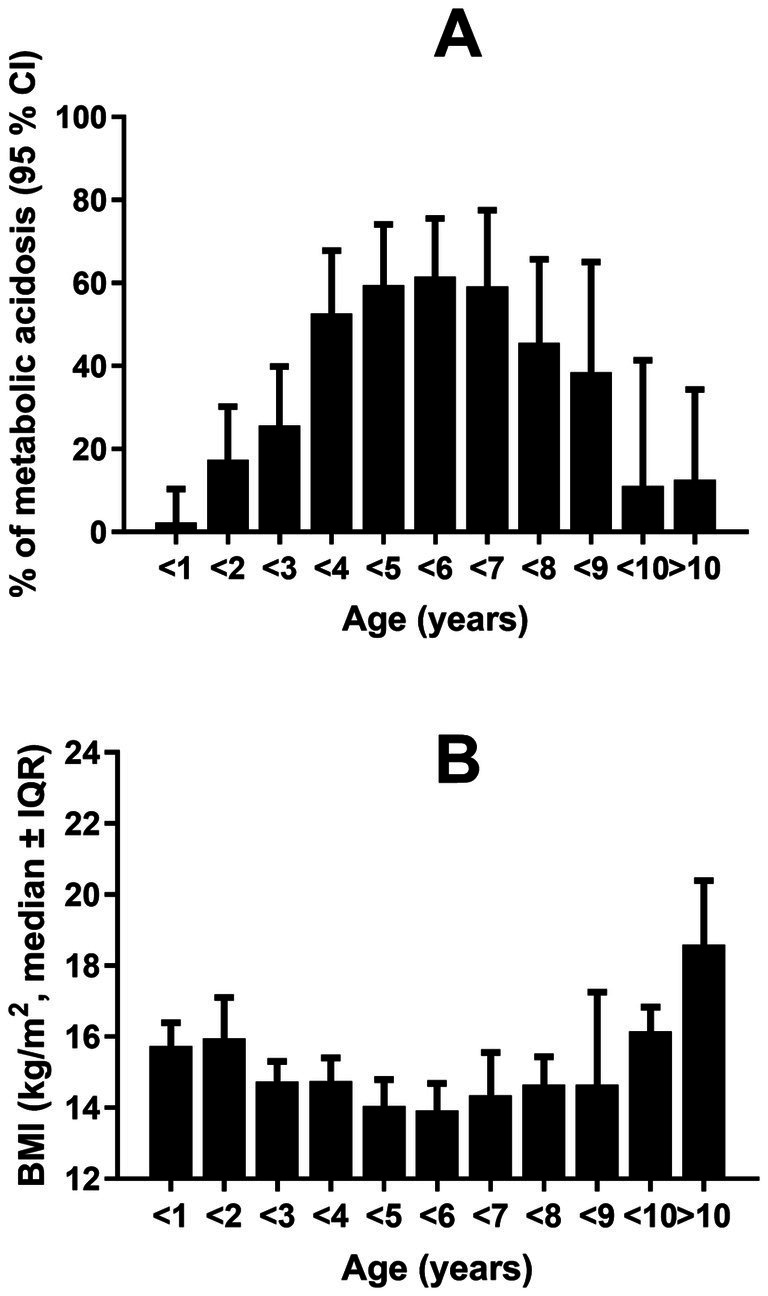
**(A)** Prevalence of metabolic acidosis in children across different age groups. Error bars represent 95% confidence intervals calculated using the Wilson/Brown method. **(B)** Body mass index (BMI) across age groups presented as median with interquartile range (IQR). The association between BMI and the prevalence of metabolic acidosis was assessed using Pearson’s correlation.

Children with MA were significantly older than those without MA (4.94 ± 2.14 vs. 3.97 ± 3.41 years; *p* = 0.002). The highest prevalence of MA (61.5%, CI 45.9–75.5) was observed in children aged 5–6 years (Figure 1). No gender difference was found (*p* = 0.356). MA was less frequent in children with diarrhea (0.9% vs. 31.8%, *p* < 0.001) but more frequent in those with complete oral intake cessation (56.4% vs. 32.2%, *p* < 0.001) and a higher degree of dehydration (*p* < 0.001).

### 3.4 Anthropometric factors

Children with MA had significantly lower BMI compared with those without MA (14.15; IQR 13.4–15.5 vs. 15.5; IQR 14.23–16.98, *p* < 0.001; Table 1). The lowest BMI (13.92 kg/m^2^, CI 13.72–14.68) and the highest prevalence of MA were observed in children aged 5–6 years (Figure 1). BMI-SDS was also lower in the MA group (−0.96; IQR − 1.34 to −0.23 vs. –0.57; IQR − 1.13 to 0.3, *p* = 0.004). Although children in the MA group were taller and heavier (*p* < 0.001), the height-SDS and weight-SDS did not differ significantly between the groups.

### 3.5 Acid–base balance

As expected, children with MA had significantly lower pH and HCO₃^−^ levels. They also showed lower partial pressure of carbon dioxide (pCO₂), base excess, and higher anion gap values (all with *p* < 0.001; Table 1).

### 3.6 Biochemical associations

MA was associated with lower serum potassium, calcium, phosphorus, and glucose levels, and with higher serum urea, creatinine, uric acid, albumin, and *β*-hydroxybutyrate concentrations (Table 1).

### 3.7 Predictors of metabolic acidosis

In multivariate logistic regression, the degree of dehydration (ordinal: mild = 1, moderate = 2, and severe = 3; *p* < 0.001), absence of diarrhea (*p* < 0.001), low BMI (*p* = 0.024), and zero oral intake (*p* = 0.034) were independent predictors of MA (Table 2). The association between BMI and acid–base status remained consistent across alternative definitions of metabolic acidosis and when the acid–base parameters were analyzed as continuous variables. Similar results were obtained after adjusting BMI for the estimated dehydration severity (Supplementary Tables S1–S4).

**Table 2:** Multivariate logistic regression model evaluating the association between BMI and metabolic acidosis.

Dependent variable	Independent variable	OR (95% CI)	*p*-value
Metabolic acidosis (pH < 7.36 and HCO₃^−^ < 22), *n* = 319	Degree of dehydration	3.92 (2.27–6.76)	<0.001
	Diarrhoea	0.03 (0.003–0.20)	<0.001
	BMI (log10)	0.004 (0.00004–0.48)	0.024
	Zero oral intake	1.79 (1.05–3.07)	0.034

Multivariate logistic regression analyses were performed using stepwise selection to assess the association between body mass index (BMI; log10-transformed) and metabolic acidosis defined by pH and bicarbonate cut-offs. Odds ratios (ORs) with 95% confidence intervals (CIs) are presented. Model was adjusted for the degree of dehydration, the presence of diarrhea, and zero oral intake at admission. BMI – body mass index, HCO₃^−^ – serum bicarbonate, OR – odds ratio, CI – confidence interval.

## 4 Discussion

In this study, metabolic acidosis was identified in one-third (33.3%) of children hospitalized for dehydration with reduced oral intake. Independent risk factors for MA included dehydration severity, absence of diarrhea, and lower BMI.

### 4.1 Pathophysiological mechanisms

Two mechanisms commonly underlie MA in acutely ill children, namely (1) ketone accumulation due to fasting-induced catabolism and (2) HCO₃^−^ loss through diarrhea. In the study cohort, 89.7% of children exhibited an increased anion gap accompanied by elevated ketone levels, indicating that metabolic acidosis secondary to reduced energy intake was the predominant mechanism. This finding is further supported by the very low prevalence of diarrhea in MA cases (0.9%) compared with non-MA cases (31.8%).

### 4.2 Known risk factors

Previous studies have shown that dehydration severity and younger age increase MA risk (6). This study confirmed the first but not the second association. In the study cohort, MA was most frequent (≥50%) in children aged 3–7 years and least frequent (<20%) in those <2 or >9 years. Although the prevalence of MA varied across age groups, the relationship between age and MA was non-linear (Figure 1), which may explain why age was not independently associated with MA in multivariate analyses. BMI, identified as an independent risk factor, may also contribute to this age-dependent pattern.

### 4.3 BMI as a risk factor

This study identified BMI as an independent predictor of metabolic acidosis. A low median BMI (<15 kg/m^2^) in children aged 2–9 years was associated with a higher prevalence of MA (>20%; Figure 1). Higher BMI likely reflects greater overall energy reserves, including muscle and fat mass, which are important determinants of metabolic resilience and capacity to maintain energy homeostasis during acute illness. Reduced energy stores may predispose to earlier onset of catabolism and acid–base disturbances.

These findings align with recently published data from a similar pediatric cohort, demonstrating that low BMI was independently associated with hypoglycemia in children hospitalized with dehydration and vomiting (11). Taken together, these observations support the concept that lower BMI may reflect reduced metabolic reserve, predisposing children to broader metabolic vulnerability during acute illness.

Importantly, body weight was measured at admission, when dehydration-related fluid loss may artificially lower BMI values. To address this potential bias, this study repeated all analyses using BMI adjusted for the estimated degree of dehydration. The association between lower BMI and metabolic acidosis remained significant across all models, suggesting that the observed relationship cannot be explained solely by dehydration-induced weight loss.

### 4.4 Other contributing factors

The absence of diarrhea was another independent risk factor. While diarrhea is a recognized cause of non-anion-gap metabolic acidosis due to bicarbonate loss, most MA cases in this study were associated with elevated anion gaps, indicating a different pathophysiology. Children with predominant vomiting often developed diarrhea later, after rehydration and resolution of MA. Those with profuse diarrhea were typically referred directly to specialized infectious disease wards and thus are underrepresented in this cohort.

### 4.5 Strengths and limitations

Strengths of this study include a relatively large sample size, detailed biochemical characterization, standardized anthropometric assessment, and the application of multivariate regression analyses with multiple sensitivity checks confirming the robustness of the findings.

This study has a retrospective, single-center design conducted in a tertiary-care hospital, where children with more clinically severe presentations are more likely to be admitted. As a result, the findings may not be fully generalizable to children with mild dehydration managed in outpatient or primary care settings.

The study population was also selective. Children with predominantly diarrheal dehydration were underrepresented, as patients with vomiting and reduced oral intake were more frequently hospitalized in this setting. This may have influenced the observed association between absence of diarrhea and MA, and should be considered when interpreting this finding.

The definition of MA (pH < 7.36 and HCO₃^−^ < 22 mmol/L) reflects local laboratory reference ranges. Although slightly broader than commonly used pediatric thresholds, sensitivity analyses using stricter combined definitions, bicarbonate-only cutoffs, and continuous acid–base parameters yielded consistent results, suggesting that the observed associations are not dependent on the specific diagnostic threshold.

Finally, body weight was measured at admission, when dehydration-related fluid loss may have influenced BMI values. Pre-dehydration or post-rehydration weights were not available. Although adjustment for estimated dehydration severity yielded consistent results, the residual measurement bias cannot be entirely excluded.

## 5 Conclusion

In addition to the known association with dehydration severity, this study identified low BMI and the absence of diarrhea as independent risk factors for metabolic acidosis in children with acute dehydration. In particular, among children aged 2–9 years, in whom a physiologically lower BMI may predispose to the development of metabolic acidosis, clinicians should be more alert when managing dehydration and vomiting in this age group.

## Data Availability

The raw data supporting the conclusions of this article will be made available by the authors, without undue reservation.
