# Supplementary material for: Low BMI increases the risk of metabolic acidosis in children with dehydration
Source: Front Nutr. 2026 May 25;13:1737321. doi: 10.3389/fnut.2026.1737321 (PMC13243093; doi:10.3389/fnut.2026.1737321)
Supplement: Supplementary file 1 [file Data_Sheet_1.PDF]

Supplementary material

Supplementary Table S1. Multivariate logistic regression models evaluating the association between BMI (log10-transformed) and metabolic acidosis defined using alternative criteria

Dependent variable	Independent variable	OR (95% CI)	p-value
**MA (pH <7.35 & HCO_3^- <22)**	Degree of dehydration	3.30 (1.92–5.69)	<0.001
	Diarrhoea	0.03 (0.004–0.23)	<0.001
	**BMI (log10)**	0.002 (0.00001–0.23)	0.010
	Zero oral intake	1.73 (1.01–2.95)	0.046
**MA (pH <7.35 & HCO_3^- <20)**	Degree of dehydration	3.43 (1.98–5.95)	<0.001
	Diarrhoea	0.03 (0.004–0.24)	<0.001
	**BMI (log10)**	0.001 (0.000005–0.11)	0.005
	Zero oral intake	1.78 (1.04–3.06)	0.036
**MA (HCO_3^- <22)**	**BMI (log10)**	0.0001 (0.0000009–0.034)	0.001
	Diarrhoea	0.32 (0.15–0.69)	0.004
**MA (HCO_3^- <20)**	**BMI (log10)**	0.001 (0.0000009–0.034)	0.002
	Degree of dehydration	2.12 (1.27–3.53)	0.004

Legend: Multivariate logistic regression analyses were performed using stepwise selection to assess the association between body mass index (BMI; log10-transformed) and metabolic acidosis (MA) defined by different pH and bicarbonate cut-offs. Odds ratios (ORs) with 95% confidence intervals (CIs) are presented. All models were adjusted for degree of dehydration, presence of diarrhoea, and zero oral intake at admission. n = 319 for all models

Abbreviations: MA – metabolic acidosis, BMI – body mass index, HCO_3^- – serum bicarbonate, OR – odds ratio, CI – confidence interval

Supplementary Table S2. Multivariate linear regression models assessing the association between BMI (log10-transformed) and continuous acid–base parameters

Dependent variable	Independent variable	B (95% CI)	p-value
**pH**	Degree of dehydration	−0.040 (−0.055 to −0.024)	<0.001
	Diarrhoea	0.044 (0.023 to 0.064)	<0.001
	**BMI (log10)**	0.145 (0.020 to 0.270)	0.023
	Zero oral intake	−0.019 (−0.035 to −0.002)	0.027
**HCO_3^-**	Degree of dehydration	−1.98 (−2.85 to −1.11)	<0.001
	Diarrhoea	2.38 (1.23 to 3.53)	<0.001
	**BMI (log10)**	15.40 (8.27 to 22.52)	<0.001
	Zero oral intake	−1.42 (−2.35 to −0.48)	0.003
**Anion gap**	Degree of dehydration	2.52 (1.40 to 3.64)	<0.001
	Zero oral intake	2.52 (1.31 to 3.73)	<0.001
	**Diarrhoea**	−2.82 (−4.30 to −1.33)	<0.001
	BMI (log10)	−12.32 (−21.52 to −3.12)	0.009

Legend: Multivariate linear regression analyses using stepwise selection were conducted to evaluate the relationship between BMI (log10-transformed) and continuous acid–base parameters (pH, serum bicarbonate, and anion gap). Unstandardized regression coefficients (B) with 95% confidence intervals (CIs) are reported. Models were adjusted for degree of dehydration, presence of diarrhoea, and zero oral intake at admission. n = 319 for all models.

Abbreviations: BMI – body mass index, HCO_3^- – serum bicarbonate, B – unstandardized regression coefficient, CI – confidence interval

Supplementary Table S3. Multivariate logistic regression models evaluating dehydration-adjusted BMI (AdjBMI; log10-transformed) and alternative definitions of metabolic acidosis

Dependent variable	Independent variable	OR (95% CI)	p-value
**MA (pH <7.36 & HCO_3^- <22)**	Degree of dehydration	4.20 (2.44–7.22)	<0.001
	Diarrhoea	0.03 (0.003–0.20)	<0.001
	**AdjBMI (log10)**	0.004 (0.00004–0.48)	0.024
	Zero oral intake	1.79 (1.05–3.07)	0.034
**MA (pH <7.35 & HCO_3^- <22)**	Degree of dehydration	3.57 (2.08–6.14)	<0.001
	Diarrhoea	0.03 (0.004–0.23)	<0.001
	**AdjBMI (log10)**	0.002 (0.00001–0.23)	0.010
	Zero oral intake	1.73 (1.01–2.95)	0.046
**MA (pH <7.35 & HCO_3^- <20)**	Degree of dehydration	3.76 (2.17–6.50)	<0.001
	Diarrhoea	0.03 (0.004–0.24)	<0.001
	**AdjBMI (log10)**	0.001 (0.000005–0.11)	0.005
	Zero oral intake	1.78 (1.04–3.06)	0.036
**MA (HCO_3^- <22)**	**AdjBMI (log10)**	0.0002 (0.000001–0.042)	0.002
	Diarrhoea	0.31 (0.14–0.67)	0.003
**MA (HCO_3^- <20)**	**AdjBMI (log10)**	0.001 (0.00001–0.068)	0.002
	Degree of dehydration	2.31 (1.39–3.84)	0.001

Legend:
Multivariate logistic regression analyses with stepwise selection were performed to examine the association between dehydration-adjusted BMI (AdjBMI; log10-transformed) and metabolic acidosis defined using alternative criteria. Odds ratios (ORs) with 95% confidence intervals (CIs) are presented. All models were adjusted for degree of dehydration, presence of diarrhoea, and zero oral intake at admission. n = 319 for all models.

Abbreviations: MA – metabolic acidosis, AdjBMI – dehydration-adjusted body mass index, HCO_3^- – serum bicarbonate, OR – odds ratio, CI – confidence interval

Supplementary Table S4. Multivariate linear regression models assessing dehydration-adjusted BMI (AdjBMI; log10-transformed) and continuous acid–base parameters

Dependent variable	Independent variable	B (95% CI)	p-value
**pH**	Degree of dehydration	−0.041 (−0.056 to −0.026)	<0.001
	Diarrhoea	0.044 (0.023 to 0.064)	<0.001
	**AdjBMI (log10)**	0.145 (0.020 to 0.270)	0.023
	Zero oral intake	−0.019 (−0.035 to −0.002)	0.027
**HCO_3^-**	Degree of dehydration	−2.17 (−3.03 to −1.32)	<0.001
	Diarrhoea	2.38 (1.23 to 3.53)	<0.001
	**AdjBMI (log10)**	15.40 (8.27 to 22.52)	<0.001
	Zero oral intake	−1.42 (−2.35 to −0.48)	0.003
**Anion gap**	Degree of dehydration	2.68 (1.57 to 3.78)	<0.001
	Zero oral intake	2.52 (1.31 to 3.73)	<0.001
	**Diarrhoea**	−2.82 (−4.30 to −1.33)	<0.001
	AdjBMI (log10)	−12.32 (−21.52 to −3.12)	0.009

Legend: Multivariate linear regression analyses using stepwise selection were conducted to evaluate the relationship between dehydration-adjusted BMI (AdjBMI; log10-transformed) and continuous acid–base parameters (pH, serum bicarbonate, and anion gap). Unstandardized regression coefficients (B) with 95% confidence intervals (CIs) are reported. Models were adjusted for degree of dehydration, presence of diarrhoea, and zero oral intake at admission. n = 319 for all models.

Abbreviations: AdjBMI – dehydration-adjusted body mass index, HCO_3^- – serum bicarbonate, B – unstandardized regression coefficient, CI – confidence interval
